# Analysis of protective effects of *Rosa Roxburghii* Tratt fruit polyphenols on lipopolysaccharide‐induced acute lung injury through network pharmacology and metabolomics

**DOI:** 10.1002/fsn3.3019

**Published:** 2022-08-25

**Authors:** Li Tang, Shuo Zhang, Min Zhang, Peng‐Jiao Wang, Gui‐You Liang, Xiu‐Li Gao

**Affiliations:** ^1^ School of Basic Medical Sciences & State Key Laboratory of Functions and Applications of Medicinal Plants Guizhou Medical University Guiyang China; ^2^ Microbiology and Biochemical Pharmaceutical Engineering Research Center of Guizhou Provincial Department of Education Guizhou Medical University Guiyang China; ^3^ School of Ethnic Medicine Guizhou Minzu University Guiyang China; ^4^ Translational Medicine Research Center Guizhou Medical University Guiyang China

**Keywords:** acute lung injury, amino acid metabolism, metabolomics, network pharmacology, *Rosa Roxburghii* Tratt fruit polyphenols, sphingolipid metabolism

## Abstract

Acute lung injury (ALI) is a respiratory disease with high morbidity and mortality rates and is the primary cause of death in children and the elderly around the world. The use of Chinese foods in the complementary and alternative treatment of ALI has attracted more and more attention. This study aimed to explore the anti‐ALI activity of Chinese functional foods *Rosa roxburghii* Tratt fruit polyphenols (RRTP). RRTP was administered to lipopolysaccharide‐induced ALI mice, and its protective effects were comprehensively evaluated by lung histopathological examination, wet/dry (W/D) ratio, and cytokine production. Metabolomics analysis was used to identify the differential metabolites and metabolic pathways in plasma, and molecular docking and systemic biology‐based network pharmacology assay were performed to explore the active components and potential therapeutic targets. The results indicated that RRTP significantly attenuated the severity of pathological changes and pulmonary capillary permeability. Furthermore, RRTP limited the increase in tumor necrosis factor alpha (TNF‐α), interleukin 1β (IL‐1β), and interleukin 6 (IL‐6) levels and the decrease in interleukin 10 (IL‐10) levels in ALI mice. Metabolomics studies revealed that RRTP markedly affected 19 different metabolites, three amino acid metabolism pathways, and sphingolipid metabolism. Moreover, network pharmacology identified AKT1 (AKT serine/threonine kinase 1), TP53, IL‐6, VEGFA (vascular endothelial growth factor A), and TNF (tumor necrosis factor) as the most promising target proteins, while quercetin, luteolin, and kaempferol were the core active components of RRTP. This study investigated the complex mechanisms of RRTP against ALI for the first time, and provided a foundation for the application of RRTP as a functional food, facilitating the research of nutritional food additives for the adjuvant treatment of ALI.

## Abstract 2

This study revealed the protective effects and mechanism of RRTP against ALI for the first time and clarified the potential therapeutic targets of RRTP on ALI. This study provided a breakthrough for the development and demonstration of RRTP as a functional food, or the research of nutritional food additives for the adjuvant treatment of ALI.

## 1 INTRODUCTION

Acute lung injury (ALI) is a respiratory disease with high mortality rates and is characterized by the severe lung inflammation, injury of endothelial and epithelial cells, inflammatory cell infiltration, and pulmonary edema (Burnham et al., 2014; Thille et al., 2013). A retrospective study involving 146,058 ICU (intensive care unit) patients younger than 18 years from 2007 to 2016 indicated that the incidence and mortality rates of ALI were 1.8% and 20%, respectively (Killien et al., 2019). It is the primary cause of death in the elderly and children around the world, with more than 10 million children dying from ALI each year (Salam et al., 2015).

Modern pharmacological studies revealed that changes in amino acid metabolism might reflect immune responses to tissue damage and repair in lung inflammation (Jones et al., 2016). Furthermore, the relationship between sphingolipids and pulmonary diseases has been confirmed (Navarrete et al., 2017), and sphingolipid metabolism provides new therapeutic targets for lung infection and ALI (Yang & Uhlig, 2011).

In addition to lung‐protective ventilation strategies and glucocorticoids as preventive interventions or treatment, there is growing attention to the use of functional foods as complementary and supplementary therapies to treat ALI. According to the in vitro and in vivo research results, many natural products contained in functional foods have a variety of anti‐inflammatory activity and lung‐protective effects. These products, including polyphenols, flavonoids, terpenoids, and alkaloids, have been proposed for the treatment of ALI (He et al., 2020).

*Rosa roxburghii* Tratt fruit (RRT), an underutilized functional food source, belongs to the Rosaceae family. Numerous phytochemical studies have demonstrated its high content in vitamin C, polyphenols, polysaccharides, organic acids, and other natural active ingredients. It is widely used in the treatment of vitamin C deficiency, inflammation, aging, diabetes, immune dysfunction, hypertension, atherosclerosis, and malignant tumors (Fu et al., 2020; Yang et al., 2020; Zhang et al., 2001). RRT polyphenols (RRTP), including tannins, flavonoids, phenolic acids, coumarins and lignans, are the second major active ingredients of RRT fruit besides ascorbic acid. RRTP exerts antioxidant, antibacterial, anti‐infective, anti‐inflammatory, and immunity‐enhancing properties and are the high‐quality food resources with great value for development and utilization (Wang, 2018; Xu et al., 2019). Human clinical tests indicated that a diet with suitable plant foods containing bioactive components such as polyphenols could alleviate inflammation (Guo et al., 2009). Although RRTP has pharmacodynamic potential in preventing ALI, its anti‐ALI efficacy and mechanisms remain unclear.

**FIGURE 1:**
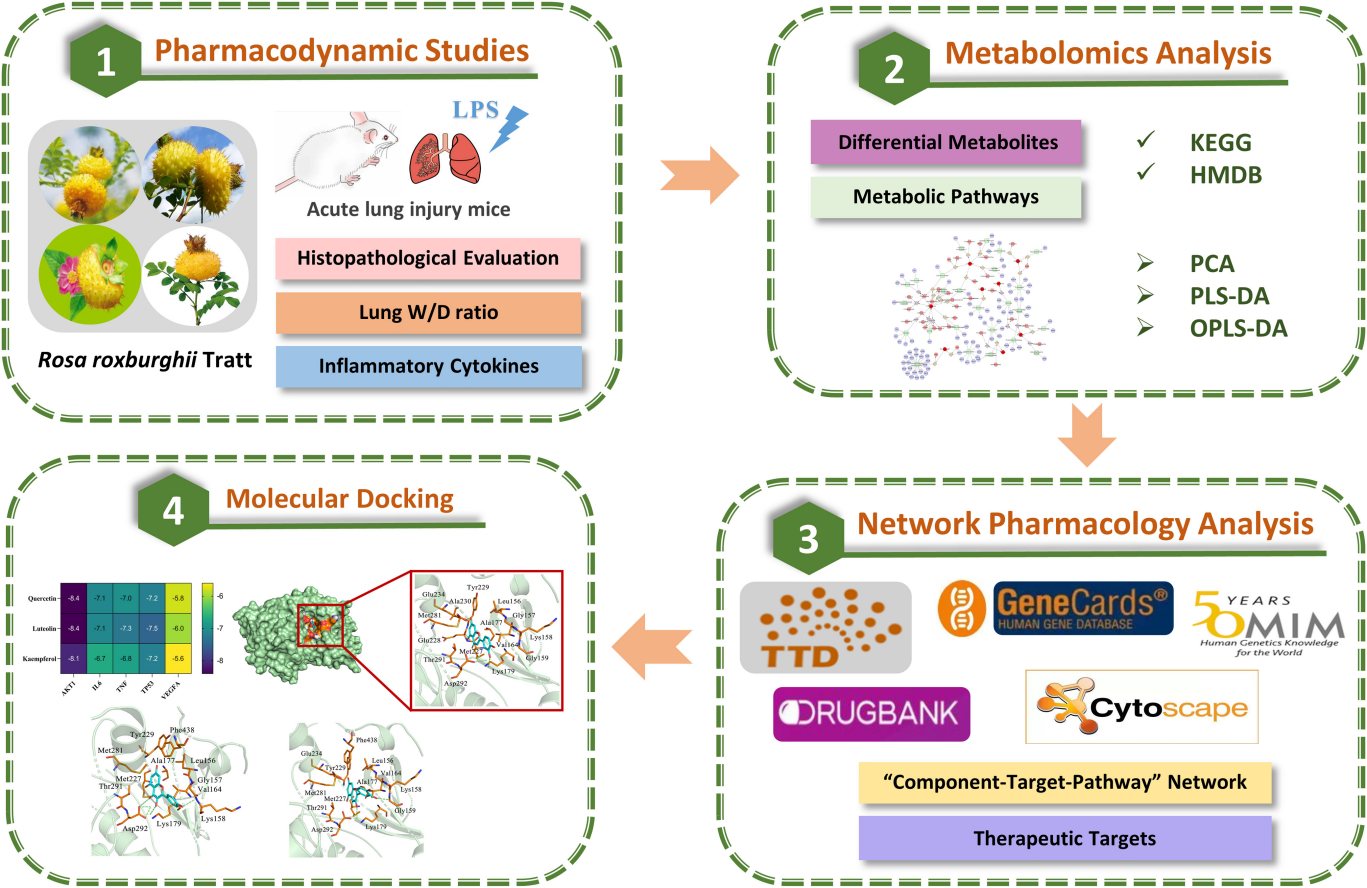
The research flow diagram of anti‐ALI mechanism of RRTP

This study applied metabolomics, systemic biology‐based network pharmacology assay, and molecular docking to explore the active components, potential targets, and pathways of RRTP against ALI for the first time. The symptoms of lipopolysaccharide (LPS)‐induced ALI mice after intervention with RRTP were explored. The pharmacodynamics results showed that RRTP had protective effects on ALI. Moreover, metabolomics analysis demonstrated that RRTP regulated phenylalanine, tyrosine, and tryptophan biosynthesis, phenylalanine metabolism, sphingolipid metabolism, and tryptophan metabolism in ALI mice. This study also identified the docking interactions between RRTP active components and core therapeutic targets, and verified the underlying mechanisms of RRTP against ALI. The research flow diagram of the anti‐ALI mechanisms of RRTP is displayed in Figure 1.

## 2 MATERIALS AND METHODS

### 2.1 Reagents

*Rosa Roxburghii* Tratt, collected from Guizhou Longshan Xiangfang Food Co. Ltd (Guizhou, China), were authenticated by Professor Xiuli Gao (Guizhou Medical University, Guiyang, China). Voucher specimens (no. 202136210) were deposited in the Microbiology and Biochemical Pharmaceutical Engineering Research Center of Guizhou Medical University. Lipopolysaccharide (*Escherichia coli serotype* O55:B5, Sigma‐Aldrich). Dexamethasone sodium phosphate injection (DEX) was purchased from Suicheng Pharmaceutical Co., Ltd. (5 mg/ml, 22,009,281, Zhengzhou, China). Mice TNF‐α, IL‐10, IL‐6, and IL‐1β enzyme‐linked immunosorbent assay (ELISA) kits were obtained from Shenzhen NeoBioscience Co., Ltd. (China). Formic acid (UPLC grade, 99% purity), methanol and acetonitrile (UPLC grade) were obtained from Merck (Darmstadt, Germany). All other chemicals and reagents were of analytical grade.

### 2.2 Isolation and preparation of RRTP

The extraction process was carried out according to the literature (Wang, 2018), and some modifications were made based on our previous research. A certain amount of RRT was mixed with 80% ethanol (1:10, W/V), and ultrasonic extraction was performed at 50°C and 400 W (power) twice. After ethanol was recovered from the filtrate, ethyl acetate was used for extraction. Finally, ethyl acetate was recovered and the solution was concentrated at 1.5 g/ml.

### 2.3 Animals and LPS‐induced ALI

A total of 45 specific pathogen free (SPF) male BALB/c mice, weighing 18–22 g (Changsha Tianqin Biotechnology Co., Ltd., Hunan, China) (License no. SCXK [xiang] 2019‐0013), were kept in a standard animal laboratory with regulated temperature (25 ± 1°C) and humidity (60 ± 5%), with a 12/12 h light/dark cycle and free access to water and food. All of the animal experiments were reviewed and approved by the Animal Ethics Committee of the institution (license no. SYXK [qian] 2018‐0001) and were performed strictly according to the Guidelines for the Animal Care and Use of Guizhou Medical University.

All animals were acclimatized for 7 days, and 40 mice with temperature fluctuations less than 0.5°C were selected. The animals were randomly divided into 4 groups (*n* = 10 each): (1) the normal group (10 ml/kg/d physiological saline by gavage for 7 days), (2) the LPS‐induced model group (10 ml/kg/d physiological saline by gavage for 7 days), (3) the dexamethasone group (DEX, 10 ml/kg/d physiological saline by gavage for the first 4 days, followed by 2 mg/kg DEX by intraperitoneal injection for the next 3 days), and (4) the RRTP group (RRTP, 10 ml/kg/d RRTP extract by gavage for 7 days). The body temperature of the mice was measured daily over the last 3 days of the experiment.

All mice were anesthetized with 10% chloral hydrate by intraperitoneal injection after 0.5 h of the final administration. The oral cavity was opened, and the tongue was gently pulled out with tweezers. LPS (2 mg/kg) was inhaled through the posterior wall of the oropharynx while the nostrils were pinched for 30 s. The normal group was given the same amount of physiological saline, instead of LPS. Finally, the mice were sacrificed 24 h after the addition of LPS. Blood samples were collected in 2 ml heparinized anticoagulant tubes, and lung tissues were harvested. The plasma was centrifuged at 3500 rpm for 10 min at 4°C and stored at −80°C for further analysis. The superior lobe of the right lung was immediately washed with cold phosphate‐buffered saline (PBS) and stored in 10% paraformaldehyde for histopathological examination using hematoxylin and eosin (H&E) staining.

### 2.4 Measurement of lung wet/dry (W/D) ratio

After cold PBS cleaning, the inferior lobe of the right lung was weighed for wet weight and dried in an oven at 60°C for 72 h. The W/D ratio was calculated according to the formula: wet weight/dry weight * 100%.

### 2.5 Measurement of cytokine concentrations

The levels of TNF‐α, IL‐6, IL‐10, and IL‐1β in plasma were quantified using ELISA kits according to the instructions.

### 2.6 Metabolite sample preparation

The plasma samples were thawed under ice water before the extraction. One hundred microliters of plasma was mixed with 300 μl 1:1 acetonitrile–methanol (V/V) to precipitate the proteins. Subsequently, the samples were subjected to vortex for 1 min, and ultrasonic extraction was performed in ice water for 10 min. Then, the samples were placed at −20°C for 30 min and centrifuged at 14,000 rpm, 4°C for 15 min. The supernatant was collected and transferred into a vial after filtering through a 0.22‐μm filter membrane for liquid chromatography–tandem spectrometry‐ion‐trap‐time‐of‐flight (LC–MS/MS‐IT‐TOF) analysis. The quality control (QC) sample used for evaluating the analytical variance of the data was mixed from each biological specimen with the same volume.

### 2.7 Chromatography and mass spectrometry conditions

The samples were detected by LC–MS/MS‐IT‐TOF (Shimadzu, Kyoto, Japan) on an Agilent Eclipse Plus C_18_ column (2.1 mm × 100 mm, 1.8 μm) with a column temperature of 40°C. The optimized mobile phase consisting of H_2_O (0.1% formic acid, A) and acetonitrile (0.1% formic acid, B) was as follows: 0–3 min, 5–35% B; 3–6 min, 35–55% B; 6–14 min, 55–80% B; 14–16 min, 80–100% B; 16–17 min, 100–100% B; 17–19 min, 100–5% B; 19–20 min, 5% B. The flow rate was maintained at 0.3 ml/min. Samples were analyzed in both positive and negative modes in an automatic pattern. The scanning range of the time‐of‐flight mass spectrometry (TOF‐MS) was 50–1000 *m/z*. The analytical conditions were as follows: ion spray voltage, +4500/−3500 V; nebulizer gas flow, 1.5 L/min; detector voltage, 1500 V; drying gas flow, 10 L/min; ion accumulation time, 20 ms; curved desolvation line (CDL) temperature, 200.0°C.

### 2.8 Data processing and analysis

Original data were processed using MZmine 2.5.3 software (Pluskal et al., 2010) and then imported into SIMCA‐P 14.1 (Umetrics AB, Umea, Sweden) software for principal component analysis (PCA) and orthogonal partial least‐squares discrimination analysis (OPLS‐DA). The significantly altered metabolites were identified using *p* < .05 in the independent *t‐test* and variable importance in projection (VIP) >1. Potential endogenous metabolites were identified (the error <10 ppm) by the KEGG (Kyoto Encyclopedia of genes and genomes) (http://www.kegg.ca/) and HMDB (The Human Metabolome Database) (http://www.hmdb.ca/). Furthermore, the metabolic pathways implicated in ALI after RRTP intervention were obtained using MetaboAnalyst 5.0, and a pathway with an impact value >0.1 was considered to be significant.

### 2.9 Collection of RRTP active components and potential targets

The active components of RRTP were collected from the literature and the traditional Chinese medicine and chemical component database (http://www.chemcpd.csdb.cn). The structures were downloaded from the PubChem database (Kim et al., 2018) (https://pubchem.ncbi.nlm.nih.gov/). As the targets are responsible for the biological functions, the potential targets for RRTP active ingredients were identified by searching SwissTargetPrediction (Daina et al., 2019) (http://www.swisstargetprediction.ch/) and the TCMSP (the traditional Chinese medicine systems pharmacology database and analysis platform) (Ru et al., 2014) (https://tcmspw.com/tcmsp.php).

### 2.10 Collection of disease‐related targets

The TTD (Therapeutic Target Database) (Hong et al., 2018) (http://db.idrblab.net/ttd/), PharmGkb (Pharmacogenomics Knowledgebase) (Whirl et al., 2012) (https://www.pharmgkb.org/), OMIM (Online Mendelian Inheritance in Man) (Joanna & Hamosh, 2017) (https://omim.org/), GeneCards (Zhang et al., 2018) (https://www.genecards.org/), and DrugBank databases (Yan et al., 2018) (https://drugbank.com/) were searched to identify the potential targets related to ALI and the four metabolisms (phenylalanine, tyrosine, and tryptophan biosynthesis; phenylalanine metabolism; sphingolipid metabolism; tryptophan metabolism). RRTP inhibits plasma metabolism disorders in ALI mice via these metabolic pathways.

### 2.11 Enrichment analysis of GO and KEGG

Targets related to RRTP activity and the four metabolic pathways were selected for Gene Ontology (GO) and KEGG enrichment assay. In addition, targets were uploaded to the DAVID (Database for Annotation, Visualization, and Integrated Discovery) (Jiao et al., 2012) (https://david.ncifcrf.gov/).

### 2.12 Network construction

The component–target–pathway interaction networks of ALI and the four metabolisms were constructed by Cytoscape 3.7.2 software, respectively.

### 2.13 PPI network analysis and molecular docking verification

String database was used to build the protein–protein interaction (PPI) network for screened targets implicated in ALI and the four metabolic pathways. Moreover, the five most important targets were used for molecular docking with the core active components of RRTP in the interaction network. The crystal structures of proteins were obtained from the RCSB (The Research Collaboratory for Structural Bioinformatics) protein database (http://www1.rcsb.org/). Hydrogenation, dehydration, ligand removal, and amino acid optimization were carried out in PyMOL software. Finally, AutoDock_Vina software was used to evaluate the minimum binding energy of RRTP ingredients and proteins, and visualize the docking models.

## 3 RESULTS

### 3.1 Effects of RRTP on pathological histology of lungs in LPS‐treated mice

**FIGURE 2:**
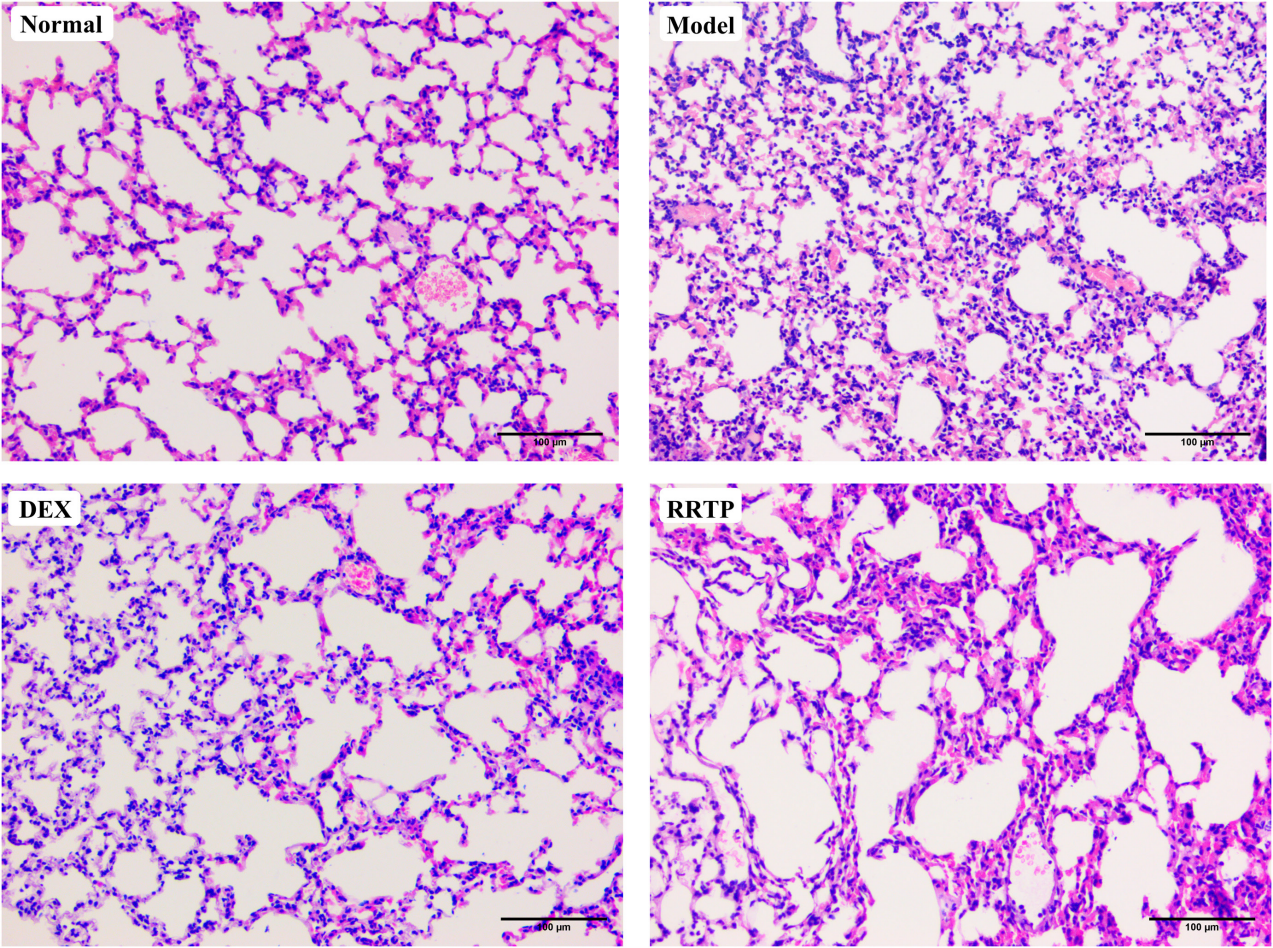
Pathological changes in the lung tissues of LPS‐induced mice. (H&E staining, original magnification ×200)

Lung tissues were subjected to evaluate morphological changes and the histological alterations after 24 h of LPS injection using H&E analysis. As depicted in Figure 2, the lung of the normal mice presented no significant morphologic damage. The results demonstrated that oropharyngeal inhalation of physiological saline caused no additional inflammation in this protocol. Furthermore, LPS‐induced model mice showed significant inflammatory cell infiltration, pulmonary congestion, and thickening of the alveolar wall, suggesting severe pathological pulmonary in ALI model mice. Remarkably, intervention with RRTP and DEX significantly attenuated the severity of the histopathological findings. These results implied that RRTP‐mediated lung‐protective effects might be involved in the maintenance of lung morphology.

### 3.2 Effects of RRTP on W/D ratio in LPS‐treated mice

**FIGURE 3:**
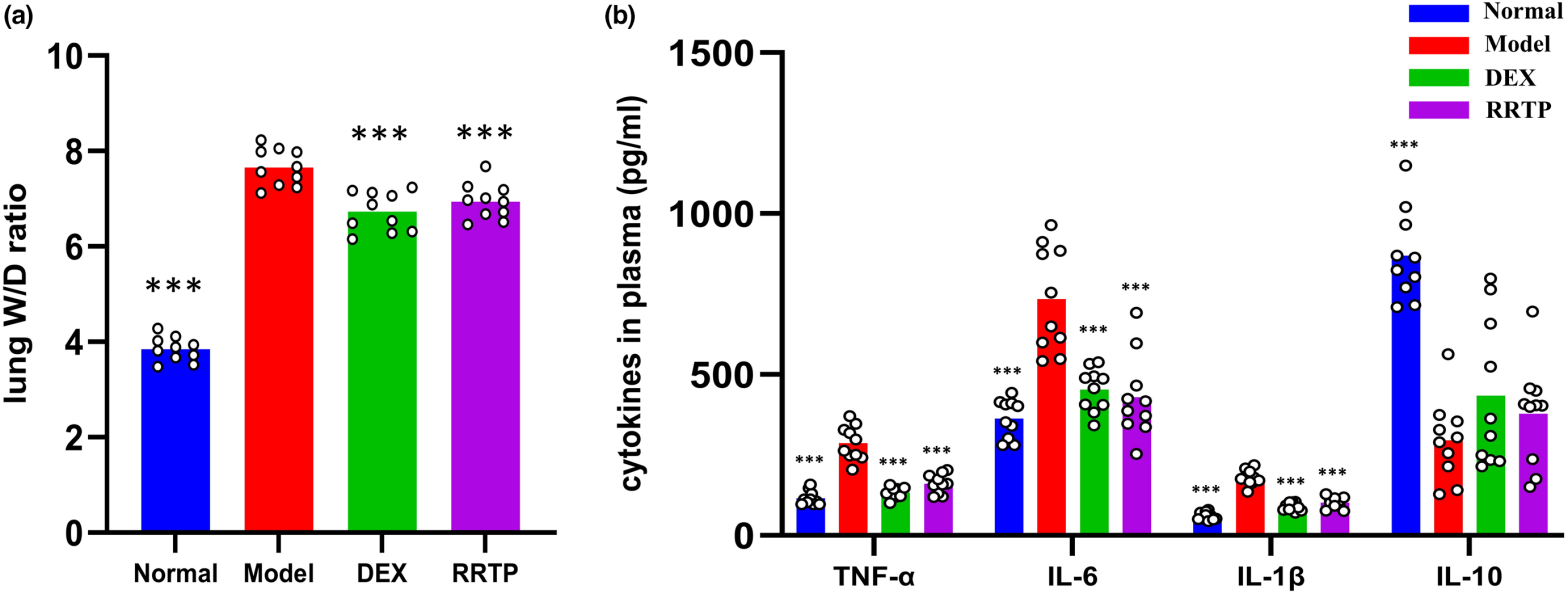
Effects of RRTP on W/D ratio in lungs and systemic inflammatory response in LPS‐induced ALI mice. (a) The W/D ratio in lungs (*n* = 10); (b) The systemic inflammatory response in plasma (*n* = 10); *Compared with the model group, ***p < .001

The W/D ratio can reflect the severity of pulmonary edema. In this study, animals exposed to LPS oropharyngeal inhalation exhibited an increase in W/D ratio compared with the normal mice. Notably, the increase in W/D ratio caused by LPS was remarkably attenuated by RRTP or DEX treatment (Figure 3a), demonstrating that RRTP and DEX could significantly reduce pulmonary capillary permeability and improve pulmonary edema in ALI mice.

### 3.3 Effects of RRTP on cytokines production in LPS‐treated mice

Pro‐inflammatory cytokines are essential in ALI progression. Consequently, to investigate the effects of RRTP on LPS‐treated ALI mice, the inflammatory cytokines were detected in plasma. As shown in Figure 3b, the cytokine levels of the model group were significantly higher than those of the normal group, except for IL‐10 (*p* < .05). Remarkably, pretreatments with RRTP and DEX restrained the LPS‐induced escalation of TNF‐α, IL‐1β, and IL‐6 and decline of IL‐10 compared with the model group. This indicated that RRTP protected ALI mice from lung inflammation by inhibiting the release of the pro‐inflammatory factors.

### 3.4 Metabolomics analysis

The LC–MS/MS‐IT‐TOF metabolic profiling data were analyzed by PCA without grouping information to estimate the overall distribution of samples and the reliability of the model. Combined with the above results, RRTP was shown to play a vital role in the intervention and regulation of abnormal pulmonary metabolic profiles in ALI mice (Figure S1). Furthermore, supervised mode OPLS‐DA was used to establish a pairwise comparison to observe the differences in metabolic profiles between the model group and the RRTP group. The reliability and fitting degree of the OPLS‐DA model was detected by the 200 permutations. The *R* ^2^ and *Q* ^2^ values were less than the original values, and the value of the intersection between the regression line of *Q* ^2^ and the vertical axis was less than zero. The results implied that the OPLS‐DA model was stable and reliable without overfitting (Figure S2). In conclusion, this model was suitable for metabolite prediction. Variables with high contribution to grouping (VIP > 1) and *p* < .05 were selected as potential biomarkers in the OPLS‐DA model.

**FIGURE 4:**
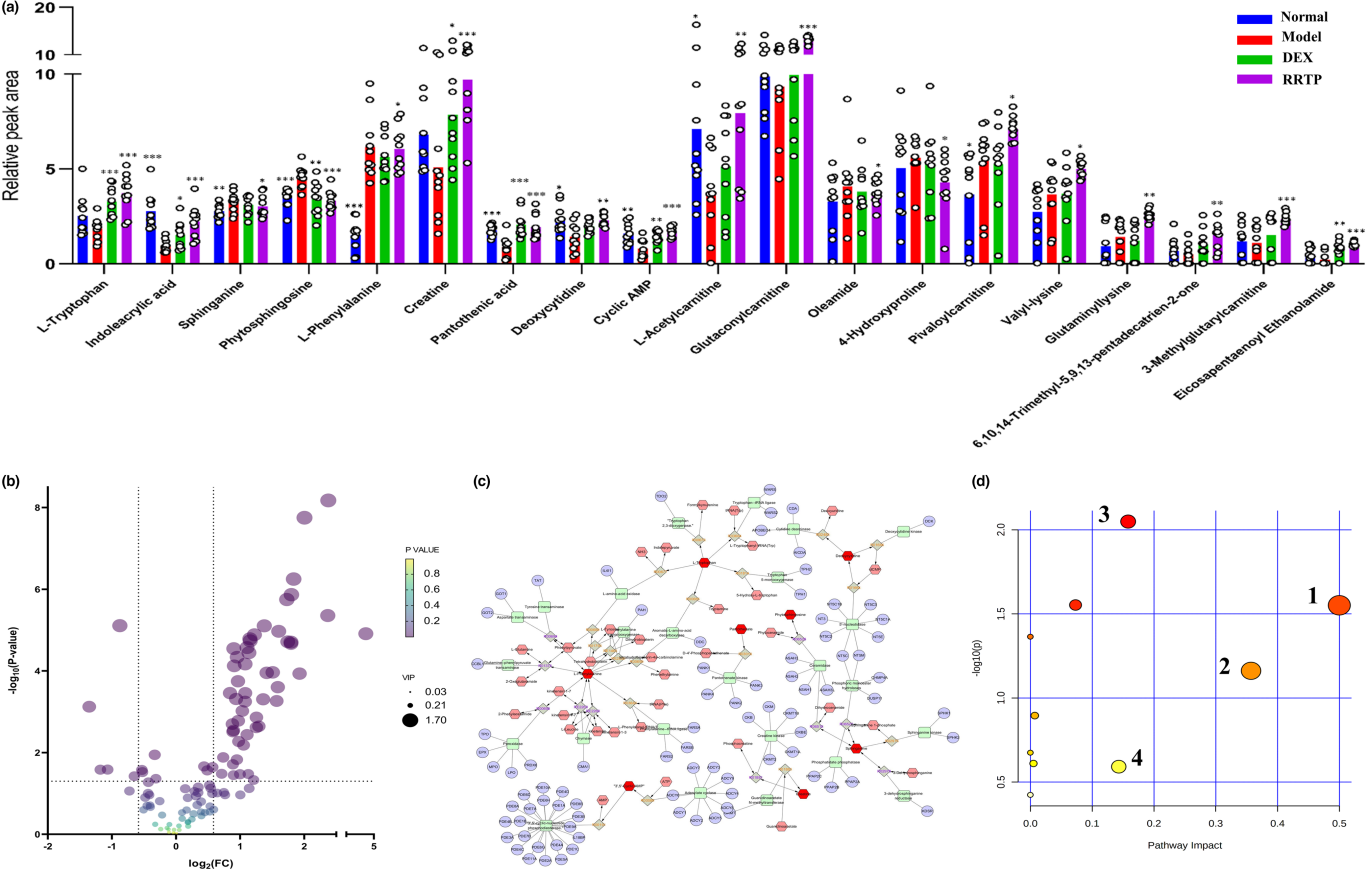
Metabolomics analysis of plasma from ALI mice. (a) Metabolites associated with LPS conditions with or without RRTP; (b) Volcano plot of the identified metabolites, the higher the ejection from the origin, the more important the variable is; (c) Metabolic network of the metabolites built using MetScape; (d) Metabolic pathways analyzed by MetaboAnalyst. 1, phenylalanine, tyrosine and tryptophan biosynthesis; 2, phenylalanine metabolism; 3, sphingolipid metabolism; 4, tryptophan metabolism; *Compared with the model group, *p < .05, **p < .01, ***p < .001

**FIGURE 5:**
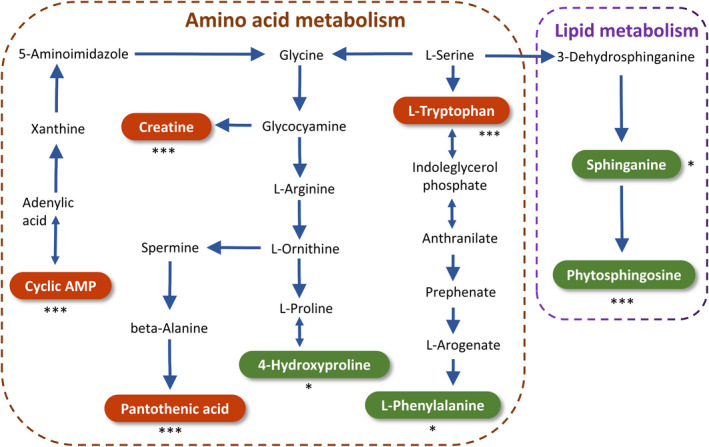
Representative metabolism showed in metabolomics analysis. Green represents pathways downregulated by RRTP whereas red represents pathways upregulated by RRTP

According to the secondary mass spectrometry information, RRTP reversed the changes of the 19 endogenous metabolites, of which 5 were downregulated and 14 were upregulated (Figure 4a,b). MetScape was used to build the compound interaction concerned with drug efficacy to further clarify the potential mechanisms of the RRTP ingredients. The metabolites were represented by red hexagons, while metabolic responses were represented by lines. The compound network, including 183 nodes and 192 edges, illustrated the internal relationship between endogenous metabolites and showed the protective effects of RRTP on ALI (Figure 4c). The results suggested that the identified metabolites were mainly related to the four metabolic pathways (Figure 4d). The representative metabolic pathways are shown in Figure 5.

### 3.5 RRTP ingredients and targets on ALI and the four significant metabolic pathways

**FIGURE 6:**
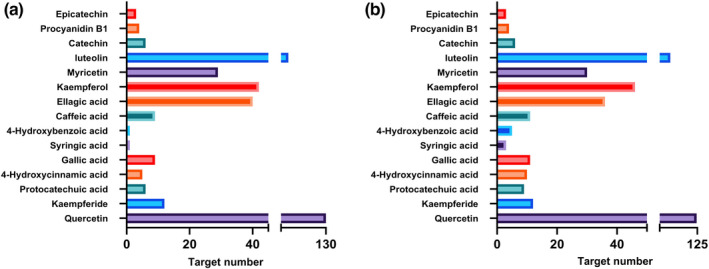
Active ingredients and targets of RRTP against ALI and the four significant metabolic pathways. (a) Active compounds of RRTP related to ALI; (b) Active ingredients of RRTP against the four significant metabolic pathways

A total of 15 active components were screened. After removing duplicates and integrating the targets of the 15 ingredients, 285 targets were confirmed. In total, 2048 targets related to ALI were obtained from GeneCards, OMIM, DrugBank, PharmGkb, and TTD databases, and a total of 182 targets were identified as the potential targets of RRTP against ALI. Furthermore, a total of 1385 targets related to the four metabolic pathways were acquired from the databases, of which 166 targets were relevant to RRTP active compounds. The number of potential targets of RRTP active compounds associated with ALI and the four significant metabolic pathways is listed in Figure 6.

### 3.6 GO and KEGG enrichment and network analysis of targets related to RRTP activity on ALI

**FIGURE 7:**
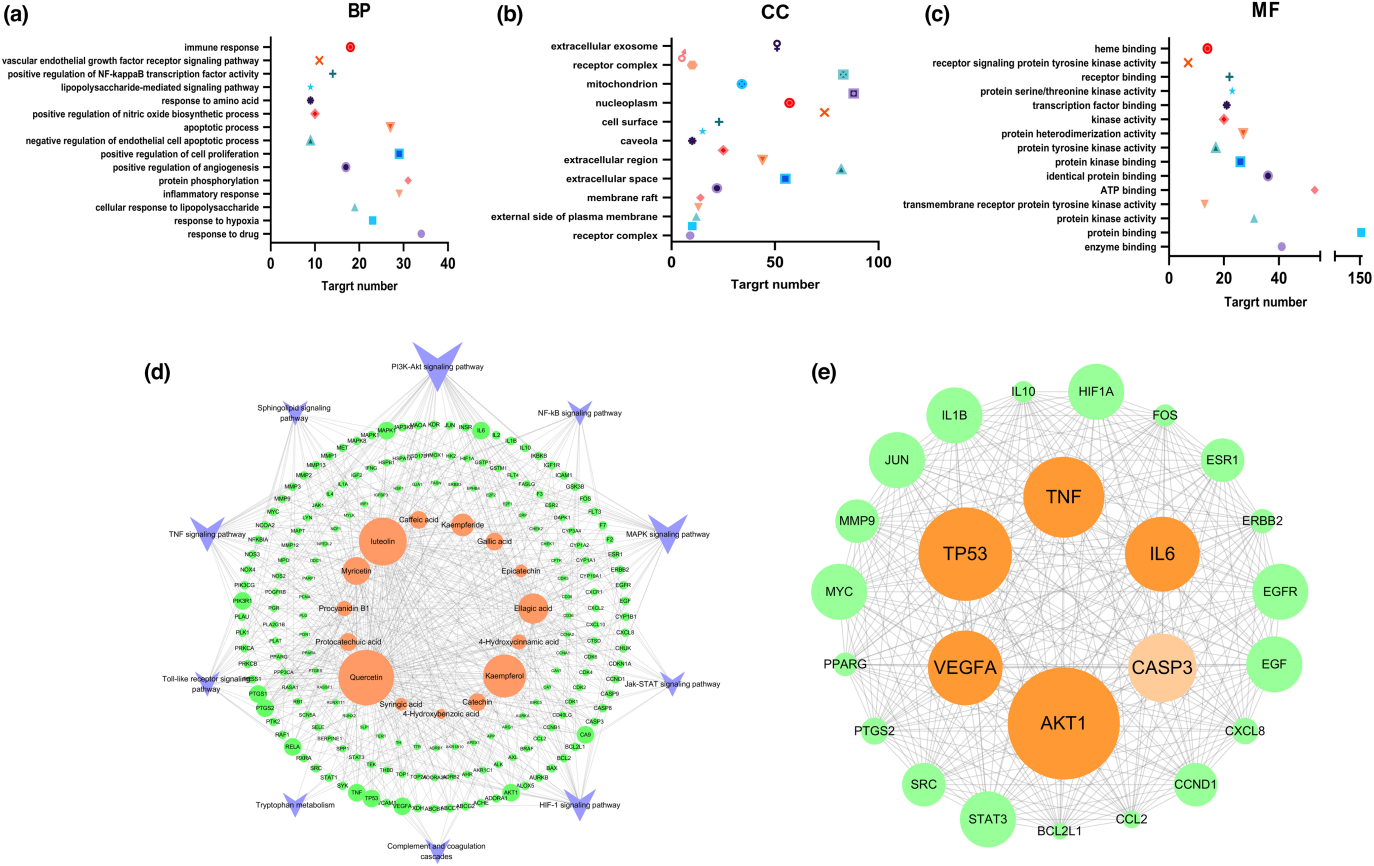
(a–c) Go enrichment analysis of potential targets for active compounds from RRTP against ALI. BP, biological process; CC, cellular components; MF, molecular function; (d) ingredient‐target‐pathway network of anti‐ALI activity of RRTP; (e) Interaction network of RRTP core target proteins involved in ALI

A total of 182 targets associated with RRTP and ALI were recorded into the DAVID database for GO and KEGG analysis (Figure 7a). In terms of biological process, the pathways were mainly involved in the apoptosis, inflammation, and oxidative stress. In the KEGG pathway analysis, signal transduction‐related pathways, amino acid metabolism‐related pathways, and immune‐related pathways were identified, which were associated with the three significant amino acid metabolism pathways and lipid metabolism pathways in the metabolomics analysis.

An ingredient–target–pathway network of RRTP implicated in the treatment of ALI was built and visualized using Cytoscape 3.7.2 (Figure 7b). This network consisted of 207 nodes and 584 edges, in which the potential relationships between targets and pathways or between active ingredients and targets were represented by edges. The size of nodes represented the degree, reflecting its important role in the treatment of ALI. The compounds with the most potential therapeutic targets, such as quercetin, luteolin, and kaempferol, could be the most critical ingredients of RRTP in protecting against ALI. About 85% of the active compounds have more than two targets, and close to 50% of the targets were regulated by more than two active compounds. These results demonstrated that RRTP active ingredients performed the effects in the entire network, which vividly reflects the complexity and completeness of the protective effects of RRTP on ALI. Furthermore, interactions between RRTP potential targets and anti‐ALI targets were evaluated using the String database. The interaction network, including 25 nodes and 299 edges, was built based on the degree value. Greater degree on targets, such as AKT1, TP53, IL‐6, VEGFA, CASP3, and TNF, indicated their essential roles in protecting against ALI (Figure 7c).

### 3.7 Pathways and network analysis of RRTP targets related to the four significant metabolic pathways

In the metabolomics study, the four metabolic pathways were identified as important pathways for RRTP to protect against ALI in mice. Therefore, the interaction network between RRTP and the four pathways was further explored in order to clarify the anti‐ALI mechanism. GO and KEGG analysis of the 166 targets related to the four significant metabolic pathways was performed using the DAVID database.

**FIGURE 8:**
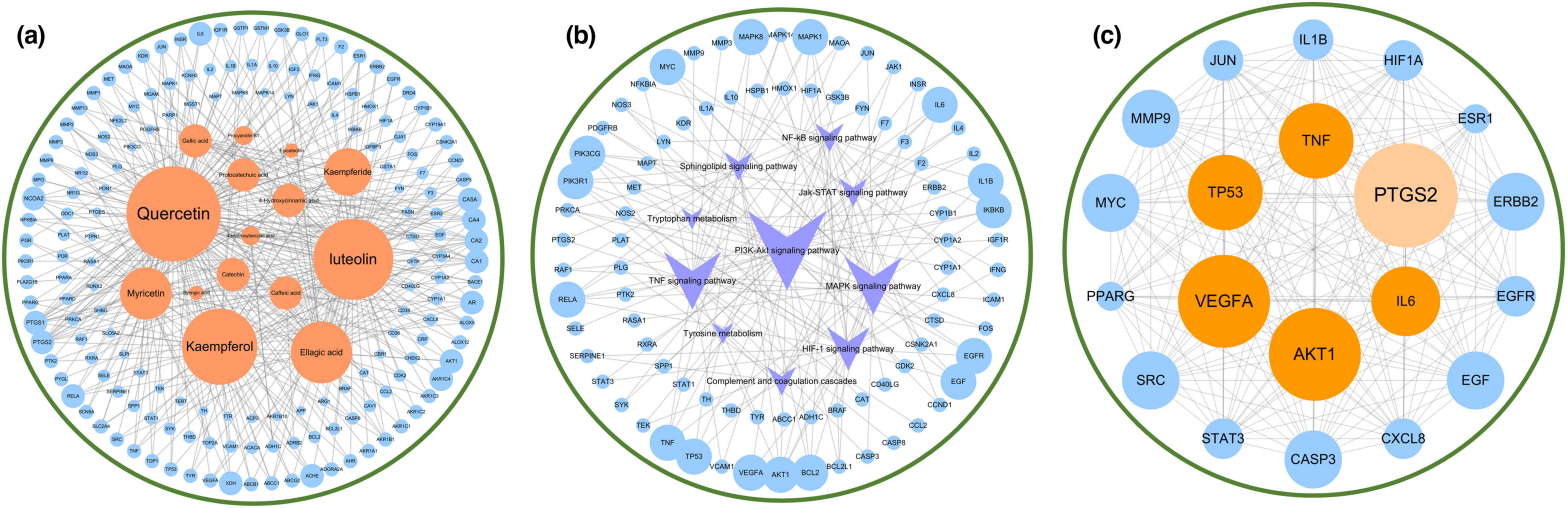
The four significant metabolism network diagrams after RRTP treatment. (a) RRTP component‐target network related to the four significant metabolism; (b) RRTP target‐pathway network related to the four significant metabolism; (c) RRTP core target proteins related to the four significant metabolism

An ingredient–target network for the four metabolic pathways was built using Cytoscape 3.7.2 based on RRTP active ingredients and predicted targets (Figure 8a). This network included 181 nodes, among which orange nodes represented active ingredients, blue nodes represented potential targets, and 392 edges represented the relationship between active compounds and targets. Quercetin, luteolin, and kaempferol regulated the most potential therapeutic targets and were considered the core active ingredients of RRTP against ALI. Moreover, a target–pathway network was constructed with 84 targets presented in blue and 10 pathways presented in purple (Figure 8b). To construct the interaction network of the four metabolic pathways and RRTP active ingredients, a degree value greater than the median was used (Figure 8c). Targets with the highest degree value played a vital role in the network.

### 3.8 RRTP active ingredients target proteins related to ALI

**FIGURE 9:**
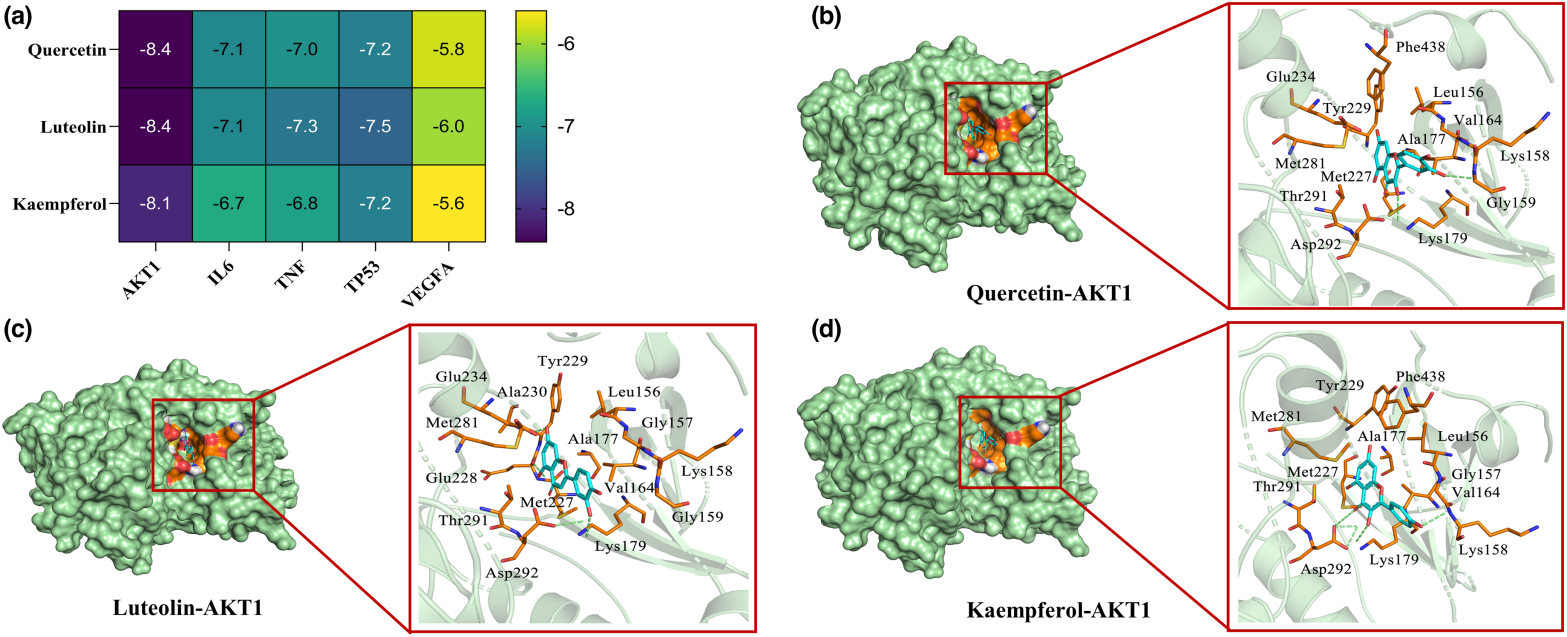
Molecular models of RRTP core active components (quercetin, luteolin, kaempferol) binding to the most promising target proteins (AKT1, TP53, IL6, VEGFA and TNF) on ALI. (a) Heat map of minimum binding energy; (b–d) The visibility graph of the model with the best docking effect

The combined experimental results of RRTP against ALI and the four important metabolic pathways affected by RRTP indicated that AKT1, TP53, IL‐6, VEGFA, and TNF were the most promising target proteins. Quercetin, luteolin, and kaempferol were the core active ingredients responsible for its efficacy against ALI. Therefore, the five most important therapeutic targets were used for docking with the three components of RRTP. The docking minimum binding energy is depicted in Figure 9a. The binding energy between target proteins and the important active components was all less than −5 kcal·mol^−1^, suggesting that the targets have a good binding activity to RRTP active ingredients. The optimal docking model of the three compounds was selected for visualization analysis (Figure 9b–d). Quercetin, luteolin, and kaempferol were predicted to bind to AKT1 by hydrogen bond and hydrophobic interactions. In summary, molecular docking analysis provided further evidence that AKT1, TP53, IL‐6, VEGFA, and TNF are underlying targets of the protective effects of RRTP on ALI.

## 4 DISCUSSION

Research in Shanghai's ICU (intensive care unit) showed that the morbidity of acute respiratory distress syndrome (ARDS) over the age of 15 was 2%, with a mortality rate of 70% (Lu et al., 2004). A study conducted by Thai researchers involving 1738 patients under the age of 15 in the pediatric ICU (PICU) from 2013 to 2016 reported the mortality and morbidity rates of 7.4% and 51.2%, respectively (Prasertsan et al., 2019). In addition, international observational research conducted during 2016–2017 in 145 PICUs from 27 countries implied that the mortality and morbidity of 23,280 patients were 3.2% and 17%, respectively (Khemani et al., 2019). Therefore, it is necessary to develop the complementary and adjunctive therapies for ALI.

The inflammatory response caused by excessive production of inflammatory mediators and cytokines is closely related to the damage to the alveolar membrane and the increase in capillary permeability (Butt et al., 2016; Meng et al., 2019). Our research suggested that RRTP significantly alleviated the severity of lung pathology in ALI mice and inhibited the increase of TNF‐α, IL‐1β, IL‐6 and the decrease of IL‐10 in lung tissues induced by LPS.

In metabolomics studies, decreased levels of sphinganine and phytosphingosine were observed in LPS‐induced ALI mice after early RRTP intervention, and RRTP significantly affected the sphinolipid metabolism. Sphingolipids are involved in several pathological features related to apoptosis, pulmonary edema, the release of inflammatory mediators, and deterioration of lung function. Elevated sphingolipids in lung tissues can lead to impaired lung function (Bismarck et al., 2012; Grosch et al., 2012; Yang et al., 2010; Yu et al., 2017). This is consistent with our results, suggesting that RRTP may improve the symptoms of ALI mice by regulating sphingolipid metabolism.

Additionally, amino acid metabolism is also one of the major metabolic pathways responsible for the inhibitive effects of RRTP against ALI mice. Amino acids, including tryptophan and phenylalanine, may play an important role in the inflammatory response against ALI. Phenylalanine can amplify the inflammatory response and aggravate the lung injury in the ALI model. The level of phenylalanine was significantly higher in nonsurvivors compared with ARDS survivors (Xu et al., 2020). Moreover, tryptophan exerted a strong protective effect on LPS‐induced ALI mice, and supplementation of tryptophan could significantly reduce the levels of ALI peroxide, neutrophil infiltration, and respiratory dysfunction (Liu et al., 2020). Interestingly, our study showed significantly higher tryptophan levels in the RRTP group, suggesting that RRTP may indirectly reduce lung injury by decreasing phenylalanine levels and increasing tryptophan levels.

Unexpectedly, in the metabolomics study, the indoleacrylic acid levels in the RRTP group were significantly higher than those of the model group. Indoleacrylic acid promotes intestinal epithelial barrier function and mitigates inflammatory responses. Stimulating indoleacrylic acid production could promote anti‐inflammatory responses and have therapeutic benefits (Wlodarska et al., 2017). Intestinal flora can decompose tryptophan into various indoles and its derivatives, including indoleacrylic acid. Therefore, we speculated that RRTP might indirectly affect the levels of tryptophan and indopropionic acid by promoting the intestinal flora of ALI mice. Elevated levels of sphingolipids and phenylalanine and decreased levels of tryptophan have been associated with numerous pulmonary diseases. Expectedly, the decrease in sphingolipids and phenylalanine and increase in tryptophan levels induced by early RRTP intervention protected against lipid metabolism and amino acid metabolism disorders and LPS‐induced lung injury.

Network pharmacology and metabolomics studies revealed that AKT1, TP53, IL‐6, VEGFA, and TNF were the most critical targets for RRTP against ALI. These targets were regulated by several active components of RRTP, such as quercetin, luteolin, kaempferol, ellagic acid, myricetin, and gallic acid.

In the inflammatory response against tissue injury and infection, excessive inflammatory cytokines such as IL‐6 and TNF‐α are activated and play a crucial part in the progression of ALI. Quercetin, the key active ingredient of RRTP, can improve the gas exchange function of ALI mice, significantly reduce the W/D ratio of lung tissues, inhibit the release of TNF‐α, IL‐6, and other inflammatory mediators, and have protective effects on LPS‐induced ALI mice (Huang et al., 2004; Xiao et al., 2007). Luteolin may play a protective role against ALI by reducing the inflammatory response and oxidative stress injury of LPS‐induced ALI mice and improving the pathological morphology of lung tissues (Zou et al., 2021). Kaempferol has also been proved to be effective in preventing ALI/ARDS by reducing the IL‐6 levels and inhibiting neutrophil infiltration (Qian et al., 2019). Additionally, high contents of ellagic acid were found in RRTP (Tan et al., 2019). As a natural polyphenol compound, ellagic acid can play a significant protective role in ALI by reducing TNF‐α and IL‐6 levels and increasing the production of IL‐10 (Guan et al., 2017). In conclusion, the effects of RRTP on ALI were mainly carried out by synergistic modulation of multiple ingredients, targets, and pathways. Therefore, RRTP can be considered as a functional food for prophylactic or adjunctive treatment of ALI and can be further developed as a potential nutritional food additive.

## 5 CONCLUSIONS

*Rosa roxburghii* Tratt fruit polyphenols (RRTP), the high‐quality food resources with great value for development and utilization, showed significant therapeutic effects against ALI. RRTP exerts anti‐ALI efficacy by regulating multiple targets and pathways through the synergistic effects of multiple active components, indicating that RRTP has obvious advantages as a supplement and in the adjuvant treatment of ALI. Our study provides a new perspective for exploring the underlying mechanisms of functional foods.

## CONFLICT OF INTEREST

The authors declared no potential conflicts of interest with respect to the research, authorship, and publication of this article.

## ETHICAL APPROVAL

The animal study was reviewed and approved by the Animal Care and Use Committee of Guizhou Medical University.

## Supporting information


Figure S1‐S2

## Data Availability

All datasets obtained and analyzed during this study are available from the corresponding author on reasonable request.
