# Supplementary material for: Analysis of protective effects of Rosa Roxburghii Tratt fruit polyphenols on lipopolysaccharide‐induced acute lung injury through network pharmacology and metabolomics
Source: Food Sci Nutr. 2022 Aug 25;10(12):4258–69. doi: 10.1002/fsn3.3019 (PMC9731534; doi:10.1002/fsn3.3019)
Supplement: Supplementary file 1 — Figure S1‐S2 [file FSN3-10-4258-s001.docx]

**Supplementary Figures**

**Analysis of protective effects of *Rosa Roxburghii* Tratt fruit polyphenols on lipopolysaccharide-induced acute lung injury through network pharmacology and metabolomics**

**Li Tang, Shuo Zhang, Min Zhang, Peng-Jiao Wang, Gui-You Liang, and Xiu-Li Gao^*^**

*** Correspondence:**

**Corresponding author**

Xiu-li Gao, Professor

State Key Laboratory of Functions and Applications of Medicinal Plants & School of Pharmacy, Guizhou Medical University, Guiyang 550025, PR China.

Tel. / fax: (+86) 0851 88416154

E-mail: gaoxl@gmc.edu.cn


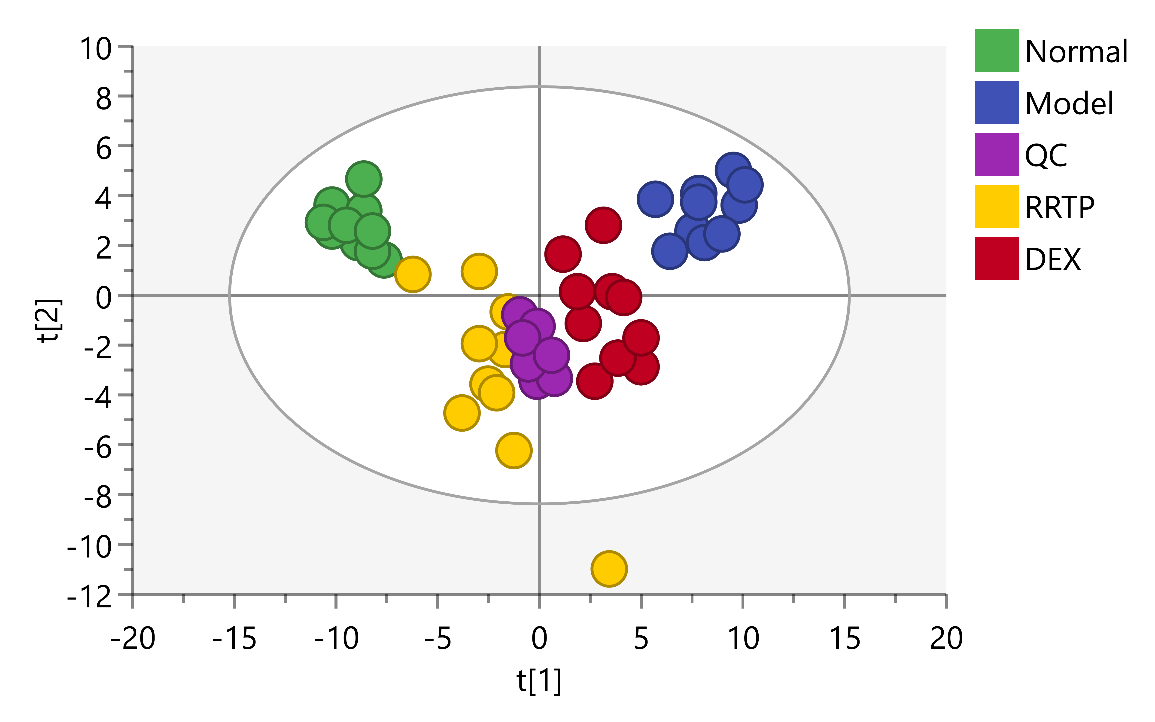


**Supplementary Figure 1.** PCA score diagrams. Without molecule selection, the metabolomics data of plasma from RRTP group were well resolved from model group mice, while the other groups showed certain degrees of separation.


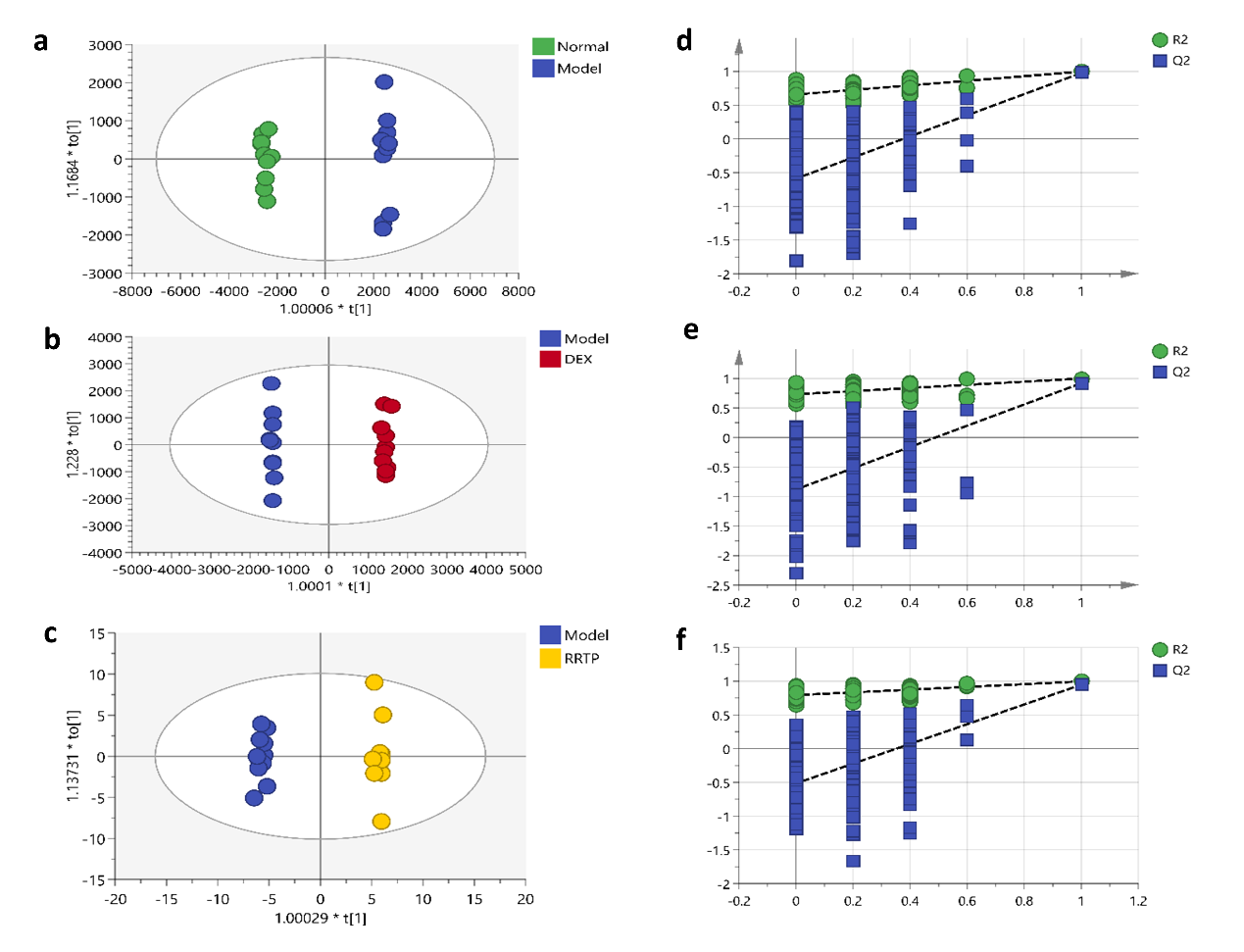


**Supplementary Figure 2.** OPLS-DA analysis. (a-c) OPLS-DA score plot. (a) Normal group vs. model group (R2Y=0.998, Q2=0.978). (b) Model group vs. DEX group (R2Y=0.999, Q2=0.92). (c) Model group vs. RRTP group (R2Y=0.996, Q2=0.948).

(d-f) Validation of OPLS-DA model by permutation test (n=200). (d) Normal group vs. model group. (b) Model group vs. DEX group. (c) Model group vs. RRTP group.
